# Metabolic Functions of Biliverdin IXβ Reductase in Redox-Regulated Hematopoietic Cell Fate

**DOI:** 10.3390/antiox12051058

**Published:** 2023-05-07

**Authors:** Wadie F. Bahou, Natalia Marchenko, Natasha M. Nesbitt

**Affiliations:** 1 Department of Medicine, School of Medicine, Stony Brook University, Stony Brook, NY 11794, USA; 2 Department of Pathology, Stony Brook University, Stony Brook, NY 11794, USA; natalia.marchenko@stonybrookmedicine.edu; 3 Blood Cell Technologies, 25 Health Sciences Drive, Stony Brook, NY 11790, USA; natasha@bloodcelltechnologies.com

**Keywords:** megakaryocyte, erythroid development, antioxidant, cytoprotection

## Abstract

Cytoprotective heme oxygenases derivatize heme to generate carbon monoxide, ferrous iron, and isomeric biliverdins, followed by rapid NAD(P)H-dependent biliverdin reduction to the antioxidant bilirubin. Recent studies have implicated biliverdin IXβ reductase (BLVRB) in a redox-regulated mechanism of hematopoietic lineage fate restricted to megakaryocyte and erythroid development, a function distinct and non-overlapping from the BLVRA (biliverdin IXα reductase) homologue. In this review, we focus on recent progress in BLVRB biochemistry and genetics, highlighting human, murine, and cell-based studies that position BLVRB-regulated redox function (or ROS accumulation) as a developmentally tuned trigger that governs megakaryocyte/erythroid lineage fate arising from hematopoietic stem cells. BLVRB crystallographic and thermodynamic studies have elucidated critical determinants of substrate utilization, redox coupling and cytoprotection, and have established that inhibitors and substrates bind within the single-Rossmann fold. These advances provide unique opportunities for the development of BLVRB-selective redox inhibitors as novel cellular targets that retain potential for therapeutic applicability in hematopoietic (and other) disorders.

## 1. Introduction

**Figure 1:**
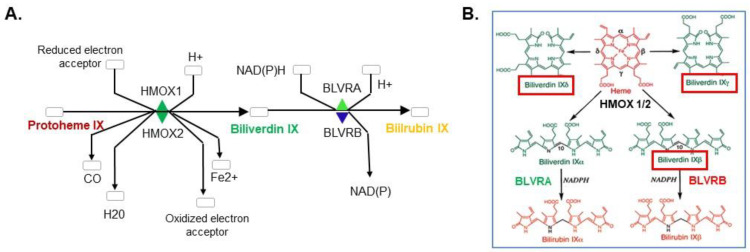
*Heme degradation pathway schema*. (**A**) Canonical pathway [Heme → Biliverdin (BV) → Bilirubin (BR)] with cofactor/product generation. (**B**) Heme-generated BV isomers highlight BLVRB substrates (BV IXβ, BV IXδ, BV IXγ, red boxes) that are distinct from those utilized by BLVRA (BV IXα); isomer-restricted bilirubins (BR IXα and BR IXβ) are shown.

Heme proteins are found in nearly all phylogeny [1], serving critical functions in gas exchange [2], as light-sensing phytochromes [3], and in electron transport [4]. Cellular accumulation of pro-oxidant free heme is regulated by two distinct heme oxygenases (HMOX1 and HMOX2), functioning in a catabolic reaction that releases carbon monoxide, free ferrous iron (Fe^2+^) and isomeric biliverdins (BV) [5,6,7]. Heme oxygenases generally utilize cytochrome P450 reductase or reduced ferredoxin as an electron source [4], and sequentially process cytotoxic heme for the generation of isomeric biliverdin (BV) and bilirubin (BR) [5]. BR functions as an antioxidant [8], and is implicated in conferring a lower cardiovascular risk [9,10,11]. The initial HMOX-mediated cleavage step could occur at any one of the four *meso* bridge carbons (designated α, β, γ, and δ, Figure 1), although regioselectivity at the α-*meso* carbon generates BV IXα as the primary isomer found in phylogenetic development (including in adult mammals) [12], to the exclusion of BV IXβ, IXγ, and IXδ. Regioselectivity is highly conserved, with a notable exception described for the *Pseudomonas aeruginosa* HMOX homologue (*hemO*; HMOX_PA), which cleaves heme for the exclusive production of BV IXβ and BV Ixδ [13,14]. This unusual cleavage is likely explained by an in-plane ~110° heme rotation within the binding pocket, resulting in repositioning of the δ-*meso* carbon where the α-*meso* carbon is situated in other heme oxygenases, the most favorable for BV IXδ generation [13,14]. A subsequent binding mode of heme rotated 180° around the α–γ *meso* axis results in the production of BV IXβ [14].

BV to BR derivatization is mediated by two non-redundant biliverdin reductases (BLVRA, biliverdin IXα reductase; and BLVRB, biliverdin IXβ reductase) that display distinct substrate specificities for isomeric BVs [15]. BLVRA retains specificity for the major BV in adults (IXα) [16,17], while BLVRB is promiscuous and catalyzes the NAD(P)H-dependent reduction of BVs IX.

β, IXδ, IXγ [15], flavins [18], and pyrroloquinoline quinones (PQQ) [19]. The relative enrichment of non-α (i.e., IXβ, IXδ, IXγ) BV/BR isomers in the fetus implies there are dichotomous function during development, and an erroneous categorization of BLVRB as a “fetal” reductase with little to no relevance to adult physiology [20], which is surprising given BLVRB’s abundance in adult organs (such as the liver) and the hematopoietic system [21]. In this review, we focus on the antioxidant and redox functions of BLVRB, focusing on the biochemical mechanisms of cytoprotection and lineage fate. For details relevant to BLVRA in human physiology, the reader should refer to excellent previously published reviews focusing on BLVRA in cytoprotection [22], metabolism [23], inflammation [24], and cancer [25].

## 2. Biochemical Features of BLVRB

### 2.1. Historical Perspective: BLVRB as a Methemoglobin Reductase

While Otto Warburg is appropriately credited for his pioneering studies dissecting the mechanisms of respiration on cancer metabolism, his initial studies from nearly 100 years ago focused on the ability of intact erythrocytes to convert O_2_-defective methemoglobin (MetHb [3+]) to O_2_-competent hemoglobin (Hb [2+]) [26]. Hemoglobin (HGB) reduction from its ferric (Fe [3+]) to ferrous (Fe [2+]) form was dramatic in restoring the oxygen-binding capacity from the inactive, oxidized form of hemoglobin (and the erythrocyte in which it was encapsulated) from a brown to a bright red color. While studying this phenomenon, methylene blue was noted to markedly stimulate the rate of methemoglobin reduction, and both the reduction of methylene blue and its stimulation of methemoglobin reduction were shown to depend on the metabolic generation of NADPH. A reductase catalyzed the transfer of electrons from NADPH to the dye (Reaction 1), and the resulting reduced methylene blue reacted directly with methemoglobin (Reaction 2), thereby explaining the dye-stimulated reduction of methemoglobin.

Reaction 1: NADPH + Methylene Blue → NADP^+^ + Reduced Methylene Blue

Reaction 2: Reduced Methylene Blue + Fe [3+]HGB → 2 Methylene Blue + Fe [2+]HGB

Although erythrocytes express BLVRB as a non-physiological methemoglobin reductase [18], subsequent work established that BLVRB functions as the methylene blue target reductase proposed by Warburg [27,28]. Indeed, BLVRB redox coupling may use methylene blue or flavins as a treatment for congenital methemoglobinemia caused by the deficiency of cytochrome b5 reductase (*CYB5A*) [18,29]. This treatment is effective both for patients with a diminished capacity to reduce methemoglobin and for those with an enhanced rate of hemoglobin oxidation. Expectedly, methylene blue inefficiently stimulates methemoglobin reduction in patients with a deficiency of glucose-6-phosphate dehydrogenase (G6PD) and its concomitant compromised capacity to generate NAD(P)H [30].

### 2.2. Structural Features of Redox Coupling

Crystal structures of both BLVRA [31] and BLVRB [15] highlight how these oxidoreductases use both NADPH and NADH as co-factors, and further refine the mechanisms of reduction and substrate selectivity. Although the trimolecular NAD(P)H/BLVRA/BVIXα structure was not solved [31], the space-filling model of the BVIXα ridge-tile conformation provides a structural basis for BLVRA’s selectivity for isomeric BV IXα. Similar to BLVRA, BLVRB is a monomeric protein with a single dinucleotide binding domain that can accommodate both NADH and NAD(P)H, although NAD(P)H more favorably. Substrates and inhibitors bind in the same pocket in close proximity to the nicotinamide moiety of NADPH. BLVRB cannot functionally accommodate BV IXα and retains specificity for the non-IXα substrates BV IXβ, IXγ, and IXδ [15,32,33]. In addition to biliverdins and methemoglobin, BLVRB catalyzes the NAD(P)H-dependent reduction of flavins, pyrroloquinoline quinone (PQQ), ferric iron, and dichlorophenolindophenol (DCPIP) [34]. The biochemical characteristics of select BLVRA and BLVRB substrates are summarized in Table 1.

BLVRB’s overall structure is similar to that of the NAD(P)^+^-dependent short chain oxidoreductases [15]. NADPH is the preferred substrate and its enhanced binding to BLVRB is mediated by salt bridges between NADP^+^’s 2′-phosphate group and the guanidinium side chains of Arg35 and Arg78, and a hydrogen bond to the hydroxyl of Thr12 [35]. The mutagenesis of Arg35 revealed that this residue plays a critical role in BLVRB’s preference for NAPDH [35]. Arg78 and Thr12 are important for BLVRB “clamping” around NAPDH [36]. Ser111, which is structurally homologous to Ser124 of UDP-galactose epimerase [37], is at the core of BLVRB’s active site [35]. To date, Ser111 has been identified as the most critical residue for BLVRB’s redox activity (see Section 3.2, vide infra), and mutagenesis impairs biliverdin reductase activity ~10-fold compared to a wild-type enzyme [33].

The reduction of substrates requires protonation and a hydride transfer, with protonation being the first step [35]. While site-directed mutagenesis has been useful for probing BLVRB’s active site, the mechanism by which the enzyme reduces its substrates is incompletely understood. Using computational modeling and site-directed mutagenesis, previous studies concluded that His153 (which could serve as a proton source for the biliverdin reductase reaction) is not important for catalytic activity, making bulk solvent the likely proton source [15,16,35]. Pre-steady state kinetic studies demonstrated that neither His153 nor Ser111 are important for hydride transfer. Molecular modeling studies to identify structural changes between apo- and holo-BLVRB revealed that loop80 (Val73 to Thr85) and loop120 (Thr110 to Gln126) block the active site, resulting in an “open” (inactive) state of the enzyme. Upon binding of NAD(P)H, these loops and the N-terminus of helix αE are repositioned to form a wall of the substrate binding pocket resulting in a conformationally “closed” (active) state of the enzyme [35]. These data suggest that this loop closure facilitates hydride transfer in a manner akin to that of the M20 loop of dihydrofolate reductase (DHFR) [38].

### 2.3. BLVRB Inhibitor Development

Predicated on in vitro, human, and murine models [21,32,39] (see Section 3.1, Section 3.2, vide infra), integrated observations suggest that the development of BLVRB-selective redox inhibitors with thermodynamically distinct chemical modifications represent logical approaches to selectively alter a regulatory pathway controlling megakaryocyte lineage expansion and human platelet counts. Strategies for inhibitor development incorporate both in silico screens of diverse compound libraries [32], and focused screens designed to reposition FDA (Food and Drug Administration)-approved drugs [40]. A comparable strategy designed to reposition FDA-approved drugs for BLVRA inhibitor development has been reported, although ineffective in ameliorating unconjugated hyperbilirubinemia in a rat model of *Ugt1a1* (UDP glucuronosyltransferase member A1) deficiency [41]. Pre-clinical studies using BLVRB-selective inhibitors have yet to be reported.

The crystal structures for BLVRB in complex with various inhibitors have been solved [15,32,40], and substrates and inhibitors are situated in the same pocket in close proximity to the nicotinamide moiety of NADPH. SiteMap analysis of the protein using Schrodinger software did not reveal evidence for an allosteric site (unpublished results). Common to all BLVRB-inhibitor complexes is an interaction between the inhibitor and Ser111; Arg174 also plays a key role in inhibitor binding [32,40]. The halogenated xanthene-based compounds erythrosin extra bluish and phloxine B have been shown to inhibit BLVRB’s flavin reductase, DCPIP reductase, and biliverdin reductase activities, with inhibition constants (K_i_) for FMN of ~0.7 μM (erythrosin extra bluish) and 1.8 μM (phloxine B) [32,33]. However, due to the size and non-drug-like properties of these compounds, they are not likely to be ideal candidates for further development as drugs. Nevertheless, information from structural studies using these compounds will likely provide insights into the key BLVRB–inhibitor interactions needed to develop more potent and selective BLVRB inhibitors.

## 3. BLVRB Hematopoietic Cellular Effects

### 3.1. Metabolic Determinants of Hematopoietic Lineage Fate: Redox and Reactive Oxygen Species (ROS)

The bioenergetic requirements of self-renewing, quiescent hematopoietic stem cells (HSC) within the hypoxic marrow niche are distinct from those during differentiation and lineage commitment [42,43,44], coincidental with a switch to aerobic metabolism [42,43,44]. Quiescent hematopoietic stem cells utilize glycolysis for the generation of ATP, and the oxidation of glutamine plays a requisite function in the survival of pluripotent stem cells [45]. Similarly, glutamine oxidation serves as a crucial mitochondrial substrate for cancer cells [46], and has been proposed as an effector of human hematopoietic stem cell lineage specification [47]. In a similar manner, the NAD^+^/NADH ratio is also controlled by glycolytic and mitochondrial activities that are dynamically regulated during differentiation or reprogramming [48]; thus, the NAD^+^/NADH redox state may also have a role in driving pluripotent stem cell fate.

HSCs are heterogeneous, with the subset selectivity regulated by environmental stressors such as inflammation [49] and aging [50], with ancillary hematopoietic effects on cardiovascular risk [51]. Stem cell fate is associated with divergent patterns of redox activity and ROS accumulation [52], such that quiescent, non-cycling cells in the hypoxic bone marrow are typically ROS^low^, exhibit low mitochondrial potential (ΔΨμ), and are dependent on anaerobic glycolysis. This adapted phenotype contrasts with that of lineage commitment that results in a metabolically-active bioenergetic phenotype manifested by ROS^high^ subsets and the conversion to oxidative phosphorylation [52,53]. ROS accumulation promotes lineage-restricted hematopoiesis [53] and is required for MK differentiation [54], although an intracellular ROS exceeding cell antioxidant capacity promotes senescence and apoptosis [52,55], which is regulated in part by transcriptional *FoxOs* [56] or cysteines [57].

Adaptive mechanisms for cellular antioxidant handling are temporally and differentially regulated during lineage speciation. Cumulative evidence further suggests that lineage bias is differentially regulated by metabolic adaptations and substrate(s) [47], chronological age [58], or spatial orientation within the bone marrow niche [59]. Thus, the general characteristics of hierarchical lineage development continue to be refined [60]. As an additional layer of complexity, stress-based hematopoietic responses remain distinct from those in a steady state [49]. Genetic networks and functionally interactive pathways causally implicated in lineage fate remain largely uncharacterized.

### 3.2. BLVRB as a Regulator of Erythro/Megakaryocyte Lineage Speciation

The first evidence linking BLVRB redox activity to hematopoietic cell fate originated from a large-scale genetic screen of cohorts with thrombocytosis (high platelet counts) [21], in which a redox-defective mutation (BLVRB^S111L^) was genetically associated with both clonal and non-clonal disorders of thrombocytosis. As outlined above (Section 2.1, Section 2.2), the critical role of Ser111 in redox activity, substrate, and inhibitor binding has now been validated by thermodynamic [35] and co-crystallization [32,40] models. Subsequent work established that defective redox activity was associated with ROS (reactive oxygen species) mishandling using both pluripotent [61] and primary hematopoietic cells [21]. Evidence for disparate effects on erythroid and megakaryocyte (MK) development were demonstrated using collagen-based culture systems in which BLVRB overexpression caused the expansion of the erythroid compartment, while redox-defective BLVRB resulted in a significant increase in CD41^+^ (α_IIB_β_3_) megakaryocytes. Furthermore, thrombopoietin-differentiated hematopoietic (CD34^+^) stem cells acquired the CD41^+^ phenotype primarily within the ROS^high^ subset, consistent with a requisite developmental ROS signal during terminal megakaryocytopoiesis [62,63]. Since ROS-associated proplatelet formation occurs during late-stage megakaryocyte development [64], ROS accumulation as an ancillary mechanism of exaggerated platelet formation remains plausible. Defective tetrapyrrole coupling as a putative mechanism unrelated to surrogate ROS accumulation also cannot be excluded.

ROS as a priming signal for accelerated hematopoietic differentiation has been previously proposed [53]. Recent studies have established that p22^hox^-dependent NADPH oxidase activity regulates ROS production and is required for megakaryocyte differentiation [54]. Low intracellular ROS levels are found during HSC quiescence, and are regulated by the *FoxO* family of transcription factors [56]. The predominant non-mitochondrial sources for ROS generation are nicotinamide adenine dinucleotide phosphate (NADPH) oxidases (NOX). Additionally, the thiol balance also contributes to hematopoietic progenitor cell fate, as the cellular redox status can be regulated by free or protein-incorporated thiols. Despite the implication of ROS signaling in cell quiescence and lineage fate [53], there are no well-characterized ROS-regulating mutations that modulate human platelet counts. In the case of the BLVRB effect(s) on megakaryocytopoiesis, it is likely that the temporally restricted nature of BLVRB-associated ROS priming provides the developmentally regulated signal for accelerated MK differentiation. Indeed, BLVRB is expressed during a brief window restricted to early megakaryocytopoiesis, a pattern distinct from that in erythroid development where BLVRB expression is delayed, progressive, and most abundant during late erythropoietic stages [21,65]. Bilineage models of erythro/megakaryocytopoiesis fail to demonstrate megakaryocyte lineage partitioning, most consistent with a model of post-commitment megakaryocytopoiesis and not altered erythroid/megakaryocyte lineage balance [66]. Divergent expansion of erythropoiesis further suggests an erythroid function in redox-regulated bioenergetic metabolism, with possible effects on stage-specific erythropoiesis [21,67,68]. Interestingly, despite the rarity of the S^111^L redox mutation in humans, a limited cohort analysis demonstrated reciprocal effects on hemoglobin (progressive anemia) and platelet counts (exaggerated thrombocytosis) in *BLVRB^S111L^* subsets, suggesting pathogenetically relevant consequences in the modulation of human blood cell counts [21].

### 3.3. Metabolic Consequences of BLVRB Deficiency Include Defective Bioenergetics and Glutamine Shunting

**Figure 2:**
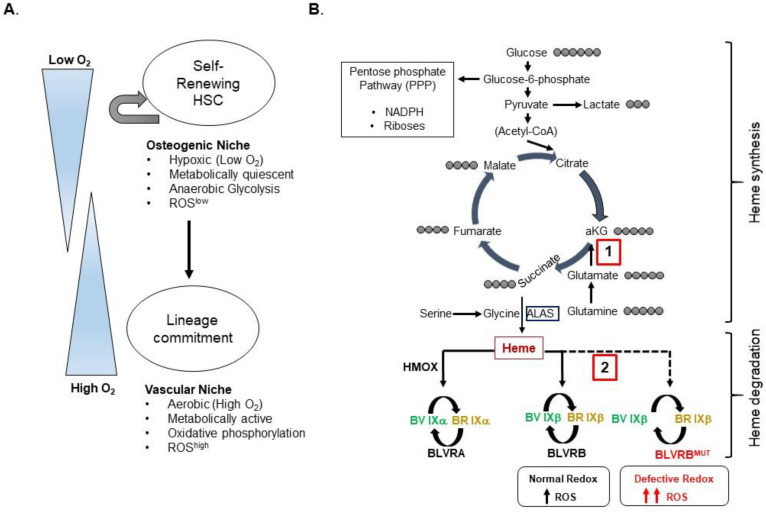
*BLVRB mechanism(s) during hematopoiesis*. (**A**) Metabolic differences between self-renewing and lineage-committed stem cells highlight adaptive differences in oxygen tension, glucose utilization, and ROS accumulation as cells transition from the normally hypoxic osteogenic niche to the oxygen-enriched vascular niche concomitant with lineage commitment. (**B**) The schema outlines critical BLVRB-associated pathways (PPP, TCA, glycolytic) that intersect with heme. Heme generation and degradation function as a linear pathway that uses glucose and glutamine carbon pools for TCA anaplerotic functions required for heme synthesis, functionally linked to downstream bilirubin (antioxidant) generation regulated by BLVRB and BLVRA (heme degradation). The parallel glucose-dependent pentose phosphate pathway provides both the NADPH reducing equivalents and riboses required for purine and pyrimidine synthesis. The rate-limiting ALAS (aminolevulinic acid synthase) reaction controls heme biosynthesis by condensation of TCA-derived succinyl CoA and glycine. Based on current models, BLVRB-associated functions in hematopoiesis have been identified in TCA glutamine uptake [1] or ROS handling [2] (highlighted by red boxes). Defective glutamine utilization would lead to attenuated heme generation, while defective or deficient BLVRB redox function (red) would result in ROS accumulation (red) as a stage-specific signal for post-commitment lineage expansion (megakaryocytopoiesis; see text for details); presence of a putative BV/BR redox cycle modeled on BV IXα/BR IXα is shown [71]. The TCA carbon atom transition map for key intermediates is delineated by gray circles.

Studies using *BLVRB*-deficient pluripotent stem cells (PSCs) imputed potentially synergistic mechanisms for BLVRB metabolic functions, coupled to defective bioenergetics and glutamate TCA (tricarboxylic acid) entry [61]. Heme synthesis and catabolism are contextually placed at the interface of bioenergetics and cellular antioxidant handling. Heme biosynthesis is initiated by the condensation of TCA-derived succinyl CoA and glycine (controlled by the rate-limiting ALAS; aminolevulinic acid synthase reaction), whose generation from serine provides 1-carbon units coupled to the folate cycle for purine and thymidine biosynthesis [69]. Thus, TCA-derived carbon using glucose or glutamine as fuels provides the anaplerotic reactions of tetrapyrrole biosynthesis [61,70]. Downstream heme metabolism directly links the heme synthetic pathway to the cytoprotective mechanisms critical to mitigating against cellular stress (Figure 2). In parallel, glucose through its G6PD (glucose-6-phosphate) reaction provides the critical NADPH reducing equivalents via the pentose phosphate pathway (PPP) for BLVRB cofactor function.

Dysregulated TCA glutamine utilization in *BLVRB*-deficient PSCs connects heme generation and degradation with anaplerotic entry into the TCA cycle. Evidence for shunted glutamine was demonstrated using isotopomeric tracings at the initial α-ketoglutarate TCA entry point. The shunted glutamine response presumably functions as an adaptive means to limit the heme generation, and results in glutamine-restricted defects in oxygen consumption under both basal and maximal respiration. Downstream effects would result in decreased heme-containing electron transport protein (ETC) proteins displaying acquired ETC defects, ROS accumulation, and functionally defective TCA cycle NAD^+^/NADH generation [72]. Previous studies have implicated glutamine-supported nucleotide biosynthesis as a metabolic requirement for hematopoietic (erythroid) lineage specification, with a transition to myelomonocytic differentiation in settings of limited glutamine [47]. These integrated observations provide a putative mechanism whereby BLVRB-regulated glutamine TCA entry during critical hematopoietic stem cell development stages could affect the lineage fate unrelated to an effect on redox-regulated antioxidant function. Since PSCs and HSCs have distinct metabolic requirements, it is likely that glutamine metabolic effects may be restricted to distinct stages of hematopoietic commitment, unrelated to the intrinsic stem cell source.

#### Glycolysis and the PPP

The BLVRB-deficient glutamine bioenergetics defect was found to be associated with enhanced glucose utilization and was largely reversible using glucose as a substrate. Energy metabolism switches from glycolysis to oxidative phosphorylation with cellular differentiation, and the preferential utilization of glycolytic pathways is a common bioenergetic feature of PSCs despite inefficient ATP generation [46,73]. Thus, the compensatory glycolytic increase evident in *BLVRB*-deficient PSCs is a presumed adaptation either for bioenergetics or for redox homeostasis through the PPP. Interestingly, dichotomous results are evident in the presence of PPP inhibition, which is responsible not only for the generation of NADPH reducing equivalents, but also the ribose-5-phosphate that is required during riboneogenesis [74]. BLVRB-deficient PSCs display enhanced sensitivity to the PPP inhibitor 6AN (6-aminonicotinamide) during early-stage embryonal body (EB) formation, whose size defines the critical first developmental stage regulating the lineage fate [75,76]. Differential effects on proliferation using glucose inhibitors were not evident. Collectively, synergistic observations place heme catabolism in a crucial pathway of glutamine-regulated bioenergetic metabolism. Interestingly, these observations further suggest that early stages of lineage potential require glutamine anaplerotic functions and an intact PPP, which partially are regulated by BLVRB activity. An extension of these observations further suggests that BLVRB inhibition represents an ancillary strategy for modulating cellular glutamine utilization, with putative interventional consequences for cancer and hematopoietic metabolism.

### 3.4. Lessons from Blvrb-Deficient Murine Models of Hematopoiesis

Computational studies from single cell murine models of hematopoiesis contextually placed *Blvrb* at the intersection of ROS-generating and stress hematopoietic pathways, with a further suggestion for lineage effects on erythroid/megakaryocyte partitioning independent of other heme degradation pathway genes [39,77]. Subsequent characterization of *Blvrb*-deficient (*Blvrb^−/−^*) mice, including necropsy at 8-, 26-, and 52-weeks, revealed no organ pathology (including hemograms and chemistries) in either male or female mice. These results are consistent with the non-requisite functions of BLVRB in humans. In contrast, differences in hematopoietic recovery were evident in *Blvr^−/−^* exposed to 5FU (5-fluoruracil) stress, with evidence that *Blvr^−/−^* mice demonstrated > 2-fold exaggerated platelet counts at peak recovery (Day 14) compared to a control. The exaggerated rebound thrombocytosis in *Blvr^−/−^* mice was unaccompanied by exaggerated RPs% (reticulated platelets), suggesting that the thrombopoietic response was not due to enhanced proplatelet formation; bone marrow histology established that exaggerated thrombopoiesis was accompanied by a ~5-fold expansion of megakaryocytes [39]. Complete hematopoietic recovery seen in *Blvr^−/−^* mice at Day 28 excluded the long-term post-stress consequences on bone marrow (BM) function. Consistent with previous data using in-vitro-differentiated megakaryocytes, exaggerated (~3-fold) expansion of the bone marrow ROS^high^CD41^+^ fraction was evident in *Blvr^−/−^* compared to *Blvrb^+/+^* mice. These results (consistent with human in vitro studies [21]) demonstrate megakaryocyte-biased reprogramming of *Blvr^−/−^* hematopoietic stem cells, exaggerated during a stress stimulus. While inflammatory stress is a well-characterized trigger for reactive thrombocytosis [78], it is also known that inflammatory cues are readily identifiable in cohorts with essential thrombocythemia [79]. Thus, stress-induced megakaryocyte bias evident in *Blvrb*-deficient mice is consistent with observations for inflammatory triggers that are identified in distinct subtypes of thrombocytosis associated with exaggerated megakaryocytopoiesis [21,80].

In contrast to enhanced megakaryocytopoiesis, *Blvr^−/−^*-stressed mice displayed erythroid repopulation defects in recovering bone marrows and spleens. *Blvr^−/−^* spleens were smaller in size, and splenic histology was most striking for the relative loss of red pulp constituents (erythrocytes and macrophages), with general preservation of the white pulp (lymphoid). A defective erythroid repopulation was caused by oxidant mishandling as documented by the identification of a 4-hydroxynonenal (4-HNE) lipid peroxidation product, a marker of oxidative stress or redox imbalance [81]. These divergent consequences on erythropoiesis and megakaryocytopoiesis would suggest differential redox and cytoprotective mechanisms of lineage speciation occurring at discrete stages of hematopoietic development. Computational models identified murine *Blvrb* as a dominant erythroid transition gene in single-cell RNA hematopoietic studies [65,82]. *Blvrb* displayed coordinate expression with erythrocyte *Rhd* (Rhesus Blood group D) antigen and reciprocal expression with megakaryocyte platelet factor 4 (*Pf4*). No correlation was seen with myeloid myeloperoxidase *(Mpo*), most consistent with a bifurcating effect restricted to erythroid/megakaryocyte lineage speciation. Among heme degradation genes (*Hmox1*, *Hmox2*, *Blvra*, *Blvrb*), only *Blvrb* displays temporally distinct expression patterns during megakaryocytopoiesis and erythropoiesis [21]; thus, *Blvrb* expression peaks during early MK development, and displays late and sustained induction during erythroid development. While the metabolic control of hematopoiesis and stem cell biology is an active area of investigation, no other redox-regulated protein with stress-associated divergent effects on megakaryocyte and erythroid fates has been described [52]. Furthermore, these collective data synergize with (and extend) a large body of research highlighting molecular differences between physiological (normal) hematopoiesis and stress hematopoiesis [83].

### 3.5. Hmox- and Blvra-Deficient Mice: Evidence for Blvrb Non-Redundancy and Divergence from Hmox/Blvra Pathways

**Figure 3:**
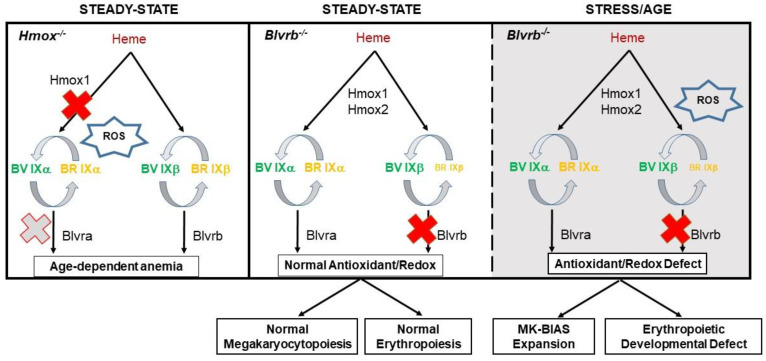
*Summary schema of hematopoietic effects evident in Hmox− and Blvrb−deficient mice*. Cytoprotective loss in *Hmox^−/−^* mice leads to an age-dependent anemia phenotype manifest by splenic red pulp loss, iron loading, loss of CD163 macrophages, and enhanced lipid peroxidation (*left panel*; note that functional *Blvra* “deficiency” may be present due to disruptive BV IXα generation). In contrast, *Blvrb^−/−^* mice maintain a normal phenotype in basal conditions despite loss of the BV/BR redox cycle (*center panel*), with unmasking of the hematopoietic phenotype in the presence of a second hit (stress, possibly age) that leads to cytoprotection mishandling (*right panel*). Blvrb redox/antioxidant functions have divergent effects in stress hematopoiesis, with megakaryocyte (MK)-biased expansion likely from an ROS-dependent developmental signal, and reciprocal erythroid loss due to defective lipid peroxidation (refer to text for details). For all panels, the cross symbol (in red) denotes gene deficiency, and relative size(s) of BV and BR impute effects on redox coupling, possibly regulated by an ROS [71].

*Blvr^−/−^* mice display phenotypic features that mirror the hematopoietic defect(s) previously described in human [84] and murine [85] *Hmox1* deficiency. These observations emphasize the critical role of an intact heme degradation pathway in both basal and stress hematopoiesis. Heme-containing proteins are present in the majority of eukaryotic cells and tissues, although erythrocyte heme encompasses ~80% of the organismal heme in mammals. Heme preponderance in erythrocytes presumably accounts for the hematopoiesis-restricted phenotype(s) demonstrable in murine models of heme degradation pathway deficiency [85,86,87]. The rarity of *HMOX1* deficiency in humans limits firm genotype/phenotype conclusions [84]. In contrast, the phenotypic characterization of murine *Hmox1^−/−^* mice demonstrates defective iron utilization, with the development of age-dependent anemia and progressive splenic fibrosis [87]. The presence of acquired and selective macrophage loss in murine *Hmox1^−/−^* deficiency is also evident, and supported by phenotypic rescue of the hematopoietic defect using macrophage-restricted bone marrow transplantation [88,89]. No comparable defects in iron utilization or macrophage loss are demonstrable in *Blvrb^−/−^* mice. While the phenotype of *Hmox1*−-deficient mice is well-established, additional information has been obtained using heterozygous *Hmox*-deficient models. Haploinsufficient *Hmox1^+/−^* mice display a progressively restricted phenotype that is manifest by a disrupted response to acute stress in stem cells and progenitors [90], with evidence for defective stress erythropoiesis in 5-FU-treated mice [86]. This stress phenotype is similar to that evident in *Blvrb^−/−^* mice, although megakaryocytopoietic effects were not described in *Hmox1^+/−^* mice [86]. The hematopoietic effects of *Hmox*- or *Blvrb*-deficient mice are summarized in Figure 3.

Recently reported *Blvra^−/−^* mice display baseline plasma oxidation differences that are different compared to wild-type controls. *Blvra^−/−^* plasma displayed (1) increased ratios of cholesterol ester hydroperoxides to cholesteryl esters (CE-OOH:CE), and (2) increased levels of α-tocopherol. Both phenotypic defects presumably result from the impaired production of the bilirubin IXα antioxidant. *Blvra^−/−^* mice also had evidence for increased red cell oxidative stress, as judged by defects in erythroid peroxiredoxin 2 activity [91]; in general, however, effects on steady-state erythropoiesis and peripheral blood cell counts appeared minimal. The role(s) of *Blvra* in stress hematopoiesis (if any) have not been described. Importantly, these collective data establish for the first time that the *Hmox* and *Blvrb* pathways are mutually exclusive stress pathways linked to the overlapping heme catabolic step (note that an intact *Hmox*/*Blvra* pathway cannot rescue the *Blvrb^−/−^* stress phenotype). These divergent and non-redundant pathways of heme degradation are consistent with the distinct and non-overlapping substrate specificities used for redox activity and reductase functions.

## 4. BLVRB and Cancer: Cellular Target and Biomarker Development

Similar to the metabolic requirements of lineage-committed hematopoietic cells, enhanced metabolic and mitochondrial activity is seen in actively proliferating cancer cells, evident by a redox imbalance and ROS accumulation that impacts the cellular viability [92]. To survive chronic oxidative stress, cancer cells evolve to activate scavenging/anti-oxidant enzymes to restore redox balance [93], thereby providing a therapeutic window for targeting novel redox targets [94]. Moreover, the effects of chemo- and radiotherapy in part are attributed to oxidative stress that causes irreversible oxidative damage and cell death, and the activation of redox-regulating pathways thought to promote resistance to such therapies [95,96,97,98]. Heme degradation is an essential pro-survival pathway that regulates redox-homeostasis in cancer cells but remains largely understudied [99]. Indeed, metabolically active cancer cells exhibit exaggerated dependence on energy production, and display an increased activity of heme-containing proteins [99,100] and/or activity [101,102]; thus, an intact heme degradation pathway is critical to mitigating against increased oxidative stress that is inherent to cancer progression and/or caused by anti-cancer therapies [103]. Conceptually, the modulation of redox-adaptation mechanisms represents a feasible strategy to eradicate cancer cells, enhance the therapeutic effects of conventional regimens, and/or prevent the onset of chemoresistance.

Evidence supporting the pathophysiological significance of BLVRB in cancer originates predominantly from unbiased proteomics and gene expression profiling. Higher BLVRB expression compared to normal tissues has been reported in numerous types of cancer, including esophageal carcinoma [104], acute lymphoblastic leukemia [105], hepatocellular carcinomas (HCC) [106,107], endometrial carcinoma [108], prostate cancer [109], pancreatic cancer [110,111], and vaginal [112] and breast cancer [113,114,115,116,117]. In endometrial carcinoma, BLVRB is highly expressed primarily at the invasive front of tumors, suggesting its potential involvement in progression and invasion [108]. Consistent with gradual expression during oncogenesis, BLVRB was shown to be associated with tumor progression in cultured cells from defined breast cancer stages and different stages of human breast cancer specimens [113]. In addition, BLVRB lymphatic expression is associated with the presence of metastases in a mouse model of breast cancer [118]. The requisite function(s) of BLVRB in carcinogenesis is supported in HCC where BLVRB overexpression promotes cell proliferation while downregulation using RNAi inhibits HCC proliferation [107].

Various studies focusing on the development of blood-based biomarkers have identified elevated BLVRB in the serum of cancer patients compared to healthy controls, including pancreatic [110,111] and breast cancer cohorts [116]. Furthermore, BLVRB has been detected in the exosomes of tumor-draining lymph comparing metastatic to non-metastatic tumors in a murine breast cancer model [118]. In addition, BLVRB has been detected in prostate cancer tissue samples (but not normal prostate) and in body fluids (urine, bladder washing samples, semen samples) isolated from prostate cancer patients. Collectively, these observations implicate BLVRB expression with tumor aggression, either as a consequence or adaptive mechanism for enhancing antioxidant defense pathways in proliferative and invasive cancers. Since cancer cells reside in hypoxic niches and rely on glutamine metabolism for survival and growth [45,46], BLVRB-selective redox inhibitors may have synergistic anti-cancer effects on both cytoprotective loss and glutamine utilization.

## 5. Conclusions and Opportunities for Future Research

BLVRB maintains a historically enigmatic role in biology, variably characterized as a flavin reductase, methemoglobin reductase, or overlooked as a “fetal” biliverdin reductase based largely on the restricted pattern(s) of isomeric BV substrates found in early fetal development. Recent renewed interest in BLVRB and the heme degradation pathway provides unique opportunities for further research, predicated on (1) initial studies in humans in which a redox mutation was causally associated with enhanced platelet counts in two distinct human disorders of thrombocytosis, additionally supported by (2) subsequent models of murine *Blvrb* deficiency that display comparable phenotypes of exaggerated proplatelet formation. Whether or not the phenotypic manifestations require an ancillary stress signal is suggested and should become more evident with future studies. Of relevance, murine models of *Hmox* deficiency highlight the importance of the heme degradation pathway in hematopoiesis. A number of parallel advances provide additional reinforcement for anticipated research progress in this area: (1) availability of BLVRB crystal structures, (2) scalable methods for isomeric BV generation and purification as BLVRB-restricted substrates, (3) availability of *Blvrb*-deficient mice, and (4) new progress on the development of BLVRB-selective redox inhibitors. Unlike BLVRA, which has a multidomain structure encompassing putative DNA binding and kinase domains [23,119,120,121], BLVRB is generally featureless and its function(s) is presumably restricted to its redox activities; distinct expression patterns and non-overlapping substrate specificities highlight critical BLVRA/BLVRB differences that mediate cellular effects. Ongoing questions relevant to hematopoietic lineage speciation will provide critical mechanistic insights that will optimally define redox-regulated cues (ROS, substrate specificity), cytoprotective functions, or synergistic effects mediated by TCA cycle bioenergetics. Since BLVRB appears dispensable for normal organ development based on murine models, BLVRB-selective inhibitors have the potential to be cellular targets for modulating hematopoietic lineage speciation or as novel cancer therapeutics.

## Figures and Tables

**Table 1:** Biochemical characteristics of select BLVRB and BLVRA substrates.

	BLVRB		BLVRA	
	Km	Reference	Km	Reference
FMN ^†^	52 μM	[12,16,18]	----	
FAD	125 μM	[12,16,18]	----	
Riboflavin	53 μM	[12,16]	----	
BV IXβ	0.3 μM	[12,16,33]	43.0–50.0 μM	[12,16]
BV IXα		----	0.8–1.0 μM	[12,16]
PQQ	2.0 μM	[19,34]	----	

^†^ Abbreviations: FMN—flavin mononucleotide; FAD—flavin adenine dinucleotide; PQQ—pyrroloquinoline quinone.
